# Genetic counselling and the intention to undergo prophylactic mastectomy: effects of a breast cancer risk assessment

**DOI:** 10.1038/sj.bjc.6600988

**Published:** 2003-05-27

**Authors:** S van Dijk, W Otten, M W Zoeteweij, D R M Timmermans, C J van Asperen, M H Breuning, R A E M Tollenaar, J Kievit

**Affiliations:** 1 Department of Medical Decision-making, Leiden University Medical Center, PO Box 9600, 2300 RC Leiden, The Netherlands; 2 Department of Clinical Genetics, Center for Human and Clinical Genetics, Section Genetic Counselling, Leiden University Medical Center; PO Box 9600, 2300 RC Leiden, The Netherlands; 3 Department of Social Medicine, Free University, De Boelelaan 859, 1082 RW Amsterdam, The Netherlands; 4 Department of Surgery, Leiden University Medical Center, Leiden University Medical Center, PO Box 9600, 2300 RC Leiden, The Netherlands

**Keywords:** genetic counselling, prophylactic mastectomy, decision making, risk perception

## Abstract

Scientific reports suggest that women at risk for familial breast cancer may benefit from prophylactic mastectomy. However, few data are available about how women decide upon this clinical option, and in particular, what role an objective risk assessment plays in this. The purpose of the present study is to assess whether this objective risk information provided in genetic counselling affects the intention for prophylactic mastectomy. Additionally, the (mediating) effects of breast cancer worry and perceived risk are investigated. A total of 241 women completed a questionnaire before and after receiving information about their familial lifetime breast cancer risk in a genetic counselling session. Path analysis showed that the objective risk information had a corrective effect on perceived risk (*β*=0.38; *P*=0.0001), whereas the amount of breast cancer worry was not influenced by the counselling session. The objective risk information did not directly affect the intention for prophylactic mastectomy. The intention was influenced by perceived risk after counselling (*β*=0.23; *P*=0.002), and by the precounselling levels of perceived risk (*β*=0.27; *P*=0.00025) and breast cancer worry (*β*=0.32; *P*=0.0001), that is, higher levels of perceived risk and breast cancer worry imply a stronger intention for prophylactic mastectomy. A personal history of breast cancer did not directly influence the intention for prophylactic mastectomy, but affected women who had undergone a mastectomy as surgical treatment were more positively inclined to have a prophylactic mastectomy than women who had had breast-conserving therapy. The impact of objective risk information on the intention for prophylactic mastectomy is limited and is mediated by perceived risk. Important determinants of the intention for prophylactic mastectomy were precounselling levels of breast cancer worry and perceived risk, suggesting that genetic counselling is only one event in the entire process of decision making. Therefore, interventions aimed at improving decision making on prophylactic mastectomy should explicitly address precounselling factors, such as personal beliefs and the psychological impact of the family medical history.

It is estimated that 5–10% of breast cancer cases are linked to a breast cancer gene mutation (Evans *et al*, 1994). Hereditary breast cancer might be suspected if the family history shows multiple cases of early-onset breast cancer, cases of male breast cancer, or cases of bilateral breast cancer, or if cases of breast and ovarian cancer occur within the same individual or family. For individuals from such families, genetic counselling is available at a family cancer clinic. Based on the family illness history, objective risk information can be provided so that clients can realistically appraise their own risk. Women with a relatively low risk may be reassured, while those with a higher risk can make informed decisions, such as deciding whether or not to undergo a prophylactic mastectomy.

The clinical option of prophylactic mastectomy remains controversial (Stefanek *et al*, 2001), although evidence for a strong protective effect of prophylactic mastectomy for women with a familial history of breast cancer has been presented (Hartmann *et al*, 1999), and, more specifically, for women with a BRCA1/2 mutation (Hartmann *et al*, 2001; Meijers-Heijboer *et al*, 2001). For instance, Meijers-Heijboer *et al* (2001) report that of 139 women with a BRCA1/2 mutation, 55% choose to undergo prophylactic mastectomy of whom none developed breast cancer, whereas 45% opted for an intensive-screening programme of whom 12% developed breast cancer within 2.9 years of follow-up. In addition, prophylactic mastectomy seems to have positive psychosocial consequences (Hatcher *et al*, 2000): high levels of psychological morbidity and anxiety before surgery decreased significantly over time after surgery, whereas in women who declined prophylactic mastectomy, a high anxiety level persisted. This suggests that women may indeed benefit from prophylactic mastectomy, although women who have to deal with surgical complications might warrant psychological help (Hopwood *et al*, 2000).

Only a few studies have reported on the decision-making process on prophylactic mastectomy of high-risk women (Stefanek *et al*, 2001). In a prospective study, Stefanek *et al* (1995) described that higher subjective risk estimates, biopsy history, and a higher level of breast cancer-related worry might be associated with the decision to have a prophylactic mastectomy. In a cross-sectional study, Meiser *et al* (2000) investigated a large sample of unaffected women, who were awaiting their initial appointment for genetic counselling. The intention to choose for prophylactic mastectomy was predicted by a very high level of breast cancer anxiety and an overestimation of the risk to develop breast cancer, whereas the objective risk of developing breast cancer did not predict intention for prophylactic mastectomy. However, hardly any data are available about the possible role of the objective risk assessment in the decision-making process.

The current study presents prospective data on whether the intention to undergo prophylactic mastectomy is influenced by (1) the objective level of risk as provided in genetic counselling; (2) pre- and postcounselling levels of breast cancer worry and perceived risk; and (3) a personal history of breast cancer. Three features distinguish the present study from other studies of prophylactic surgery decision making, that is, (a) the comparison of a pre- and postcounselling survey; (b) the inclusion of women with a history of breast cancer; and (c) the broad study population inclusive of both low- and high-risk women.

## METHODS AND MATERIALS

### Participants and procedure

Data were collected within the framework of a larger study on risk perception and decision making by women at risk for hereditary breast cancer. For the integrative study, ethical approval was obtained from the hospital's research ethics committee. Participants were at least 18 years of age with a family and/or a personal history of breast cancer who applied for genetic counselling at the Department of Clinical Genetics of the Leiden University Medical Center. Referrals for genetic counselling on breast cancer were based on current national guidelines (De Bock *et al*, 1999). In the first (and sometimes only) consult, a clinical geneticist interviewed the women applying a standard counselling protocol, and recorded their family medical history. Information was provided about the hereditary transmission of BRCA1 and BRCA2, and about surveillance, if applicable. Genetic testing was offered if there was a probability of mutation detection of about 10% or more. If sufficient medical information was available, a familial lifetime risk of developing breast cancer was estimated (Claus *et al*, 1994). Four risk categories were distinguished: (1) general population risk, that is, around 10%; (2) 10–15%; (3) 15–30%; and (4) 30% or more. The standard protocol of the first consultation did not cover any discussion about pros and cons of prophylactic mastectomy. If supplementary medical information had to be collected or if genetic testing was applied, further appointments were scheduled (53% of the women). In general, for women with a relatively low risk and who were not eligible for genetic testing, no further appointments were made (47% of the women). (Further appointments fell outside the scope of the data reported here, as all measures were conducted before and after the first consultation.)

All new referrals for breast cancer counselling from November 1998 until July 2001 were informed about this study by letter 2 weeks before their first appointment. Eligible women who returned the written consent form that accompanied the informative letter received the first questionnaire by mail prior to their first appointment. Immediately after this counselling session, a second questionnaire was sent out, irrespective whether follow-up appointments were scheduled. Reminder letters were sent, if appropriate. Women were excluded from the study if they had not received information about their familial lifetime risk during the counselling session, had lost both breasts due to previous surgery, had distant metastases, or if they had an insufficient literacy in the Dutch language.

## MEASURES

### 

#### Sociodemographic characteristics

Information on personal history of breast cancer (i.e. unaffected or affected women), surgical procedure to treat breast cancer (i.e. mastectomy or breast-conserving therapy), age, educational level, marital status, and number of children was collected.

#### Breast cancer-related worry

In both questionnaires, we assessed breast cancer-related worry with two items of the breast cancer worries scale (Lerman *et al*, 1991). These items were as follows: ‘During the last 2 weeks, how often did you worry about developing breast cancer yourself (again)?’, and ‘During the last 2 weeks, how often did your worries about breast cancer interfere with your daily activities?’ on a four-point scale ranging from 1 (almost never) to 4 (almost all the time). The mean of both items was calculated (scores ranged from 1 to 4), with higher scores indicating a higher level of breast cancer-related worry. The reliability of this scale was satisfactory (precounselling: Cronbach's *α*=0.66; postcounselling: Cronbach's *α*=0.73).

#### Perceived risk of breast cancer

Perceived risk was assessed in both questionnaires with a comprehensive scale that included various aspects of perceived risk: (1) relative perceived lifetime risk of getting breast cancer was measured with the item ‘Compared to the average Dutch woman, my risk of developing breast cancer (again) is 1 ‘very much lower’, through 4 ‘equal to’, to 7 ‘very much higher’, (2) numerical perceived lifetime risk of getting breast cancer was measured with the item ‘My risk of developing breast cancer (again) is … out of 100’, and (3) verbal risk with the item ‘Independent of my actual risk, I feel my risk of developing breast cancer (again) is 1 ‘very low’ to 7 ‘very high’. Perceived risk was measured in both the questionnaires. As the range of items varied, standardised scores of the separate items were used. The precounselling measures of perceived risk were each z-transformed. The postcounselling measures were similarly standardised also using the mean and standard deviation of the corresponding precounselling items. The mean of the three standardised items constituted the perceived risk scale. The scale had an adequate reliability (precounselling: Cronbach's *α*=0.78; postcounselling: Cronbach's *α*=0.73). Larger values on the scale indicated a higher perceived risk.

#### Intention for prophylactic mastectomy

In the second questionnaire, the intention for prophylactic mastectomy was measured with the item ‘Do you expect to decide for preventive surgery of your breasts’ on a seven-point scale ranging from 1 ‘certainly not’ to 7 ‘yes, certainly’. This item was considered as potentially confronting to women. Therefore, the intention for prophylactic mastectomy was measured only after the counselling session.

### Statistical methods

The SPSS 10.0 statistical package was used to analyse the data. Path analysis was applied to examine the research questions with several multiple regression analyses (Cohen and Cohen, 1983). In the first phase, we checked whether the objective risk provided in the counselling was related to either having had breast cancer (no or yes), or to the precounselling measures of perceived risk and breast cancer worry.

In the second phase, we applied two multiple regression analyses to assess whether there was a change in perceived risk, respectively in worry, and, if so, which factors predicted the change. In order to do so, the change scores between the pre- and postcounselling scales of (a) perceived risk and (b) worry were calculated by subtracting the precounselling value from the postcounselling value. Thus, a positive value indicated increased worry or perceived risk, and a negative value implied decreased worry or perceived risk after the counselling session. These two change scores served as outcome variables. Four predictor variables were used in each analysis: (a) having had a personal history of breast cancer, (b) the objective risk information, and the precounselling measures of (c) perceived risk, and (d) worry. The precounselling measures of perceived risk and worry were included, because the possible range of change is determined by the precounselling values. However, the interpretation of predictive effects of the precounselling measures on the change measures is hampered by the fact that the precounselling measure is a constituent part of the change score. Therefore, we will not describe the relations between the precounselling measures and the corresponding change scores in the path analysis. (However, Pearson's correlations are presented in Table 3

**Table 3:** Pearson correlation between the predictor and the outcome variables

	**Variable**	**1**	**2**	**3**	**4**	**5**	**6**
1	Breast cancer (no/yes)						
2	Perceived risk (before)	−0.162*					
3	Worry (before)	0.104	0.389***				
4	Objective risk	−0.063	0.174**	0.098			
5	Perceived risk (change)	0.187**	−0.429***	−0.132*	0.286***		
6	Worry (change)	0.122	−0.151*	−0.407***	−0.017	0.240***	
7	Intention mastectomy	0.071	0.284***	0.356***	0.158*	0.103	0.000

* *P*<0.05

** *P*<0.01

*** *P*<0.001.

.)

In the third phase, the intention for prophylactic mastectomy was predicted from all previous variables. In this phase, we also wanted to examine whether the overall model to predict intention for prophylactic mastectomy would differ between women from the highest risk category (i.e. >30%) and women in the lower risk category (i.e. ⩽30%). The choice for this dichotomy was based on the fact that only women in the highest risk category will be eligible for genetic testing as the chance to harbour a BRCA mutation must be sufficiently high. Two-way interaction variables with risk status (multiplication of centred scores) were included in the analysis (e.g. interaction between worry, precounselling and the change score, and risk status; perceived risk, precounselling and the change score, and risk status; and breast cancer history and risk status).

To check whether the observed relations in phases 1–3 would differ between affected and unaffected women, two-way interaction variables with breast cancer history were included in the analyses in a similar way as described above for risk status interactions (e.g. interaction between worry, precounselling and the change score, and breast cancer history; perceived risk, precounselling and the change score, and breast cancer history; and objective risk and breast cancer history). In addition, for affected women we examined whether the surgical procedure to treat their breast cancer served as an additional predictor in the phases 1–3 regression analyses.

For each multiple linear regression analysis, we report the extent of variance in the criterion explained by the regression (*R*^2^), the significance of the explained variance (F-test), and which predictors significantly contributed to this explained variance (*β*-weights). A *P*-value <0.05 was considered to indicate statistical significance.

## RESULTS

### Study population

Of the 454 women who met the inclusion criteria, 350 consented to participate in the study (response rate 77.1%). Of these 350, 44 women returned their informed consent just at the first counselling session, instead of mailing it beforehand. Therefore, they could not complete the precounselling questionnaire. Another three were excluded from the analysis because they did not return the precounselling questionnaire in time, whereas 13 women did not complete all items of the precounselling questionnaire. Finally, 30 women did not return the postcounselling questionnaire, and 19 women did not complete all items of the postcounselling questionnaire. This left 241 women for our analyses.

*T*-tests and chi square tests showed that the women who did not complete the pre- or the postcounselling questionnaire did not differ from women who did complete all items of both questionnaires on the measures relevant for this study (objective risk, perceived risk, breast cancer worry, intention mastectomy, personal history of breast cancer, known mutation running in the family, age, marital status, educational level). On only one variable these groups differed. Women who did not complete the pre- or postcounselling questionnaire reported having children more frequently (*P*=0.025) than women who did complete all items of both questionnaires.

#### Sociodemographic characteristics

Table 1

**Table 1:** Sociodemographic and medical variables of the study population

	n	%
Sociodemographic		
*Age* (years)		
<30	39	16.2
30–39	64	26.5
40–49	79	32.8
50+	59	24.5
*Marital status*		
Married or cohabitating	194	80.5
Not married or cohabitating	47	19.5
*Children*		
Yes	162	67.2
No	79	32.8
*Educational level*		
High school or university	95	39.4
Less than high school	146	60.6
		
Medical		
*Breast cancer history*		
Yes	73	30.3
No	168	69.7
*Objective risk* (%)		
10 (population risk)	10	4.1
10–15	22	9.1
15–30	73	30.3
>30	136	56.5
*BRCA detected in family*		
Yes	25	10.4
No	216	89.6

summarises the sociodemographic and medical variables of the study population. The mean age of the group was 41.4 years (range 19–71 years; s.d. 11.0 years). The majority of the women was married or cohabitating and had one or more children. Almost half of the women was educated to high school or university level. A small minority of the women had at least one close family member in whom a BRCA1 or BRCA2 mutation had been detected.

#### Description of the outcome and predictor variables

Of the 241 women, 168 were healthy and 73 had been treated for breast cancer (36 mastectomy, 36 breast-conserving therapy, one unknown). In the counselling session, more than half of the women (*n*=136) was classified into the highest risk category (i.e. a risk of more than 30% to develop breast cancer; see Table 1), and were consequently eligible for genetic testing. The objective risk was not related to any of the sociodemographic variables. Not surprisingly, women with a known BRCA1 or BRCA2 mutation in the family had a significantly higher objective risk, than women without a known BRCA1 or BRCA2 mutation running in the family (*P*=0.0001).

Table 2

**Table 2:** Worry and risk perception precounselling and postcounselling

	**Precounselling**	**Postcounselling**
Breast cancer-related worry (scale)	1.70 (0.65)	1.72 (0.64)
* How often did you worry about developing breast cancer yourself (again)? (1–4)*	2.01 (0.82)	2.07 (0.83)
*How often did your worries about breast cancer interfere with your daily activities? (1–4)*	1.32 (0.62)	1.32 (0.61)
		
Perceived risk (scale)	0.00 (0.83)	−0.24 (0.81)
* Compared to the average Dutch woman, my risk of developing breast cancer (again) is (1–7)*	5.83 (1.21)	5.68 (1.16)
* My risk of developing breast cancer (again) is … out of 100*	54.93 (21.31)	43.86 (21.03)
* Independent of my actual risk, I feel my risk of developing breast cancer (again) is (1–7)*	4.72 (1.15)	4.63 (1.23)

Mean score and standard deviation are given in brackets.

depicts the mean values of the individual items used to measure breast cancer worry and perceived risk. The majority of the women had no excessive breast cancer-related worry either before or after counselling (precounselling *M*=1.70; postcounselling *M*=1.72). Most women stated that they almost never or only sometimes worried about developing breast cancer (precounselling 73.8%; postcounselling 72.2%). Similarly, almost all women reported that worries about breast cancer almost never or only sometimes interfered with their daily activities (precounselling 94.2%; postcounselling 94.2%).

The perceived risk prior to and after the counselling session was high. The vast majority of the women (precounselling 89.2%; postcounselling 86.7%) thought their risk to be higher than the average Dutch woman's risk. In addition, approximately half of the women (precounselling 60.2%; postcounselling 55.2%) stated that, independent of their actual risk, they felt they had a high risk of developing breast cancer (again). Finally, most of the women perceived their numerical risk to develop breast cancer (again) to be 30% or more (precounselling 86.3%; postcounselling 74.7%).

Figure 1

**Figure 1:**
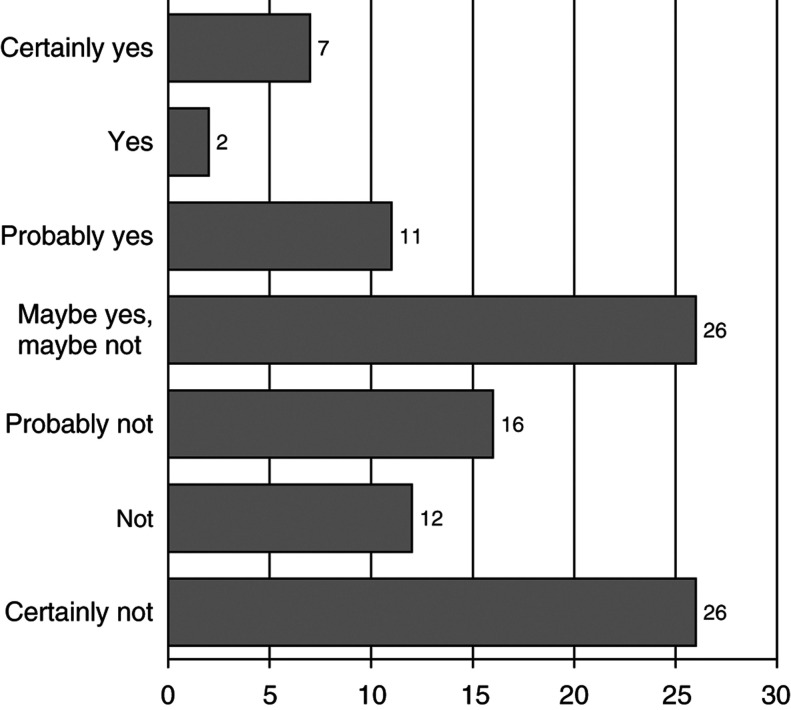
Intention to undergo prophylactic mastectomy (percentages).

displays the data on the intention to choose for prophylactic mastectomy. Overall, the majority of the women certainly or probably expected to decide against prophylactic mastectomy (54.4%), whereas 19.9% certainly or probably expected to decide for prophylactic mastectomy. A quarter of the women was undecided (25.7%).

### Description of the path analysis

**Figure 2:**
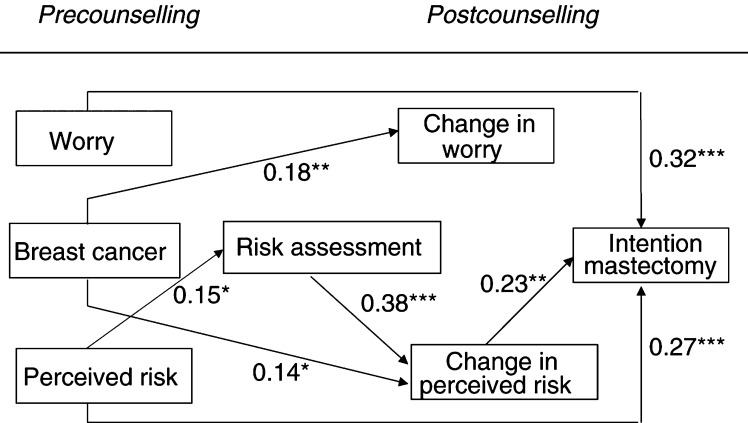
Full regression model with Beta's (^*^P<0.05, ^**^P<0.01, ^***^P<0.001).

Path analysis was applied to examine our research questions. Below, each multiple regression analysis is described separately. Table 3 shows the zero-order Pearson's correlations between the predictor and outcome variables; Figure 2

depicts the combined results.

#### Phase 1: was the objective risk information related to precounselling variables?

Neither the precounselling measure of breast cancer-related worry (*P*=0.53), nor having had a primary breast tumour (*P*=0.51) was associated with the objective risk information. However, the perceived risk women reported before the consult was positively related to their actual risk (*β*=0.15; *P*=0.037). The model was significant in explaining objective risk (F(3,237)=2.71, *P*=0.046, *R^2^*=0.033).

#### Phase 2a: Did perceived risk change after counselling and which factors predicted such a change?

Overall, perceived risk decreased after the counselling session (*constant*=−1.33; *P*=0.0001). As expected, the objective risk information influenced this change in perceived risk (*β*=0.38; *P*=0.0001). Women with a relatively low objective risk reported a lower perceived risk after counselling, whereas women with a relatively high objective risk remained at a high level of perceived risk after counselling. This means that after counselling women shifted towards a more accurate perceived risk. To illustrate this point, we looked at the numerical risk estimates that women provided. Before the counselling, 83% of the low-risk women overestimated their risk and after counselling 56% overestimated their risk. In contrast, of the high-risk women 89% correctly identified their high-risk status before and after counselling. The change in perceived risk was also predicted by having had breast cancer (*β*=0.14; *P*=0.015). Unaffected women showed a stronger decrease in perceived risk after counselling than women with a history of breast cancer. The regression model was significant in explaining the change in perceived risk (F(4,236)=29.80, *P*=0.0001, *R^2^*=0.34).

#### Phase 2b: Did the amount of breast cancer worry change after counselling and which factors predicted such a change?

Overall, breast cancer-related worry slightly increased after the counselling session (*constant*=0.56; *P*=0.001). The objective risk information, as provided in the counselling, did not influence the change in breast cancer worry after counselling (*P*=0.61). Having had a primary breast tumour (*β*=0.18; *P*=0.003) predicted the change in worry. Women who had had a primary breast tumour reported a higher level of breast cancer worry after counselling, whereas unaffected women showed no change in the amount of worry after counselling. The regression model was significant in explaining the change in breast cancer worry (F(4,236)=14.35, *P*=0.0001, *R^2^*=0.20).

#### Phase 3: Which variables predicted the intention for prophylactic mastectomy?

Precounselling levels of breast cancer worry (*β*=0.32; *P*=0.0001) and perceived risk (*β*=0.27; *P*=0.00025) both independently predicted the intention for prophylactic mastectomy. Women who had higher prior levels of breast cancer worry and/or a higher prior perceived risk reported a stronger intention to choose for prophylactic mastectomy. Intention for prophylactic mastectomy was also predicted by the change in perceived risk after the counselling session (*β*=0.23; *P*=0.002); women who shifted towards a lower perceived risk, reported a weaker intention for prophylactic mastectomy. As phase 2a showed, this change in perceived risk was influenced by the objective risk information. Although the objective risk did not have a direct effect on the intention (*P*=0.78), the objective risk information had an indirect effect by adjusting perceived risk, which in turn affected the intention.

The change in worry after the counselling session did not add to the prediction of the intention for prophylactic mastectomy (*P*=0.082). Finally, having had a primary breast tumour had no direct influence on the intention for prophylactic mastectomy (*P*=0.67). However, as shown in phase 2a, having had breast cancer influenced the change in perceived risk and, consequently, had an indirect effect on the intention for prophylactic mastectomy. The full regression model was significant in explaining the intention for prophylactic mastectomy (F(6,234)=10.99, *P*=0.0001, *R^2^*=0.22).

#### Interaction effects of risk status on intention for prophylactic mastectomy

The inclusion of the interaction variables with risk status did not affect the overall model as depicted in Figure 2, as all the same main effects remained significant at a similar level. In addition, none of the interaction variables reached significance (*P*_s_>0.102), indicating that the same model applied to low- as well as to high-risk status women.

#### Interaction effects of breast cancer history and breast cancer surgery

No effects are observed for the interaction variables with breast cancer history in the phases 1–3 regression analyses, indicating that the relations depicted in Figure 2 similarly apply to healthy and affected women.

For affected women, the kind of surgical procedure to treat their breast cancer did not predict the objective risk estimate, nor the change in perceived risk or breast cancer worry. However, the intention for prophylactic mastectomy was predicted by the kind of surgical procedure (*β*=0.22; *P*=0.040): women who had had a mastectomy showed a stronger intention to have a prophylactic mastectomy of the contralateral breast than women who had had breast-conserving surgery. Thus, for affected women the kind of surgical procedure served as an additional predictor, next to the precounselling levels of perceived risk and worry, and the change in perceived risk after counselling.

## DISCUSSION

The impact of the objective risk information provided in genetic counselling on the intention to opt for prophylactic mastectomy is relevant, but limited. First, the objective risk information had an indirect effect on the intention through the perceived risk of developing breast cancer: counselling a lower objective risk decreased the perceived risk after counselling, which related to a weaker intention to opt for prophylactic mastectomy. Second, both stronger breast cancer worry and a higher perceived risk about developing breast cancer before counselling promoted the intention for mastectomy.

The finding that perceived risk has a stronger impact on preventive intentions than objective risk is consistent with studies assessing those relations before the counselling (e.g. Stefanek *et al*, 1995; Meiser *et al*, 2000). The present study shows that the impact of the objective risk information on the intention for prophylactic mastectomy is mediated through the change in perceived risk after counselling. These results stress the importance of assessing women's perception of the risk in order to understand their decisions and behaviour regarding prophylactic mastectomy (see also Hatcher *et al*, 2000).

The present study clearly shows that the objective risk information had a corrective effect on perceived risk, but it was a moderate impact in terms of explained variance: 14% of the variance in the change of perceived risk was due to the objective risk information. This points at other factors in- or outside the counselling session that possibly affect the change of perceived risk. All in all, our results are in line with previous studies showing that genetic counselling generally improves perceived risk, but often women tend to report an inaccurate risk of developing breast cancer even at 1-year follow-up (Watson *et al*, 1999; Meiser *et al*, 2001; Meiser and Halliday, 2002).

High levels of worry and perceived risk before women approach the geneticist strongly related to the intention for prophylactic mastectomy. This supports the notion that the counselling is not the onset of deliberations regarding prophylactic mastectomy, but an element in an earlier started and ongoing process. The results even suggest that the objective risk information provided in the counselling may be a relatively small event in this process of decision making. This fits recent acknowledgements that precounselling factors like past cancer stressors are important determinants for subsequent distress and behaviour (Zakowski *et al*, 1998; Baider *et al*, 1999; Erblich *et al*, 2000; Rees *et al*, 2001). The personal experience of the counsellee, including concomitant fears and emotional beliefs, is an essential element of the counselling interaction. Only if this experience and its emotions are discussed openly and understood, will it be clear to both counsellor and counsellee what the full scale of the problem is, and to what extent objective risk assessment may or may not solve this problem.

About a third of the women who applied for genetic counselling in the present study had had a primary breast tumour. Breast cancer history had no direct impact on the intention for prophylactic mastectomy: affected and unaffected women had the same, somewhat negative, intentions. Moreover, the interaction analyses showed that the same relations applied for both healthy and affected women. This corroborates the findings of Julian-Reynier *et al* (2001) that affected women did not differ from healthy women regarding their attitude towards the acceptability of prophylactic mastectomy after multivariate adjustment. However, for affected women the kind of surgical procedure to treat their breast cancer had a direct impact: women who had undergone a mastectomy were more positively inclined towards a prophylactic mastectomy of the contralateral breast than women who had had breast-conserving therapy. Probably, uncertainty reduction and cosmetic reasons do not only apply to the decision how to treat breast cancer, but also to preventive management.

Nonetheless, in the present study breast cancer history did have an indirect effect through risk perception on the intention. Healthy women showed a stronger decrease in perceived risk than affected women, and a decreased risk perception was related to a weaker intention. Affected women were also more worried after counselling, although this did not influence the intention. In contrast, a recent study (Bish *et al*, 2002) did not find differences on perceived risk nor worry between affected and unaffected women. An explanation for the present findings is that the recurrence risk of breast cancer constitutes a possible topic in the counselling session. This might induce distress and a sense of vulnerability in affected women who may have felt relatively safe after having had breast cancer.

A few limitations of this study should be noted. First, we want to mention that a relatively large proportion of respondents, who provided written informed consent for the study, did not complete both questionnaires, mainly due to logistic problems. However, women who did not complete the questionnaires were comparable to women who did. As a consequence, we think the results are generalisable to the population of women that seek genetic counselling.

Secondly, one could view the use of intention instead of actual behaviour as a restriction. The actual use of prophylactic mastectomy will probably fall below levels of intended use (e.g. Lerman *et al*, 2000). However, the goal of the present study was to prospectively assess the impact of objective risk information on thoughts about prophylactic mastectomy for all women applying for genetic counselling for breast cancer; thus, not restricting the sample to either unaffected women or high-risk women who are eligible for genetic testing. The present sample probably covers the variety of women that seek genetic counselling now that hereditary breast cancer and genetic testing have become a topic that receives a lot of media attention. Our data suggest that for both low- and high-risk women their intention is not clearly guided by their objective risk, although only the latter women are eligible for DNA testing, and possibly prophylactic mastectomy. This points at the possibility that women at a lower risk for breast cancer may have similar desires concerning their risk management as women with a very high risk (see Bottorff *et al*, 2000). The effect of DNA-testing results on high-risk women's actual decisions regarding prophylactic mastectomy and low-risk women's risk management beliefs and behaviours will be explored in other papers.

Third, in our study we confined genetic counselling to providing information about the familial lifetime risk to develop breast cancer. This does not acknowledge the interactive features and the many other topics and goals that characterise the counselling process, which may also affect subsequent perceptions and behaviours (see Lobb *et al*, 2002). The effect of breast cancer history on worry after counselling illustrates this point: apparently, an element other than the objective risk information provided in the counselling increased the worry in affected women relative to unaffected women.

The main advantages of the present study concern the diversity of the participants, and the prospective design. Most importantly, it shows (a) the relevant, but limited impact of objective risk information on postcounselling deliberations, and (b) the major impact of precounselling factors on these deliberations. Health-care professionals should be aware of the specific limitations of counselling, and of the potential impact of women's personal experiences and beliefs concerning breast cancer. These precounselling factors should be explicitly addressed in the genetic counselling protocol, and should be a guiding element in the process of providing information.
